# Sitting Time, Physical Activity and Sleep by Work Type and Pattern—The Australian Longitudinal Study on Women’s Health

**DOI:** 10.3390/ijerph14030290

**Published:** 2017-03-10

**Authors:** Bronwyn K. Clark, Tracy L. Kolbe-Alexander, Mitch J. Duncan, Wendy Brown

**Affiliations:** 1 Cancer Prevention Research Centre, School of Public Health, The University of Queensland, Herston 4006, Australia; 2 Centre for Research on Exercise, Physical Activity and Health, School of Human Movement and Nutrition Sciences, The University of Queensland, St Lucia 4072, Australia; tracy.kolbe-alexander@usq.edu.au (T.L.K.-A.); w.brown@uq.edu.au (W.B.); 3 School of Health and Wellbeing, The University of Southern Queensland, Ipswich 4305, Australia; 4 School of Medicine & Public Health, Priority Research Centre for Physical Activity and Nutrition, Faculty of Health and Medicine, The University of Newcastle, University Drive, Callaghan, NSW 2308, Australia; Mitch.Duncan@newcastle.edu.au

**Keywords:** sleep, sitting, physical activity, women, work

## Abstract

Data from the Australian Longitudinal Study on Women’s Health were used to examine how work was associated with time spent sleeping, sitting and in physical activity (PA), in working women. Young (31–36 years; 2009) and mid-aged (59–64 years; 2010) women reported sleep (categorised as shorter ≤6 h/day and longer ≥8 h/day) and sitting time (work, transport, television, non-work computer, and other; summed for total sitting time) on the most recent work and non-work day; and moderate and vigorous PA (categorised as meeting/not meeting guidelines) in the previous week. Participants reported occupation (manager/professional; clerical/sales; trades/transport/labourer), work hours (part-time; full-time) and work pattern (shift/night; not shift/night). The odds of shorter sleep on work days was higher in both cohorts for women who worked shift or night hours. Longer sitting time on work days, made up primarily of sitting for work, was found for managers/professionals, clerical/sales and full-time workers. In the young cohort, clerical/sales workers and in the mid-aged cohort, full-time workers were less likely to meet PA guidelines. These results suggest multiple behaviour interventions tailored to work patterns and occupational category may be useful to improve the sleep, sitting and activity of working women.

## 1. Introduction

Given that most working adults spend around a third of their day at work, the occupational environment is likely to have a bearing on time spent sitting, sleeping and being active, and on subsequent health risks. Long periods spent sitting, sleep duration outside the recommended range (e.g., 7–8 h) and a lack of moderate to vigorous physical activity (MVPA) are associated with increased cardio-vascular disease risk, incident type 2 diabetes and premature mortality [1,2,3,4,5]. The duration and timing (night/day) of paid work, as well as the demands of work in terms of seated or active tasks, have been associated with overall daily sitting, activity and sleep, and with transport and leisure-time physical activity. Hence relationships between these three behaviours (sitting, sleep, physical activity) are complex and likely to be intertwined with patterns and duration of work.

Several small studies have examined associations between occupation, work hours and sitting time. One Australian study has shown that call centre workers spend significantly more time in sedentary behaviour while at work, than office and customer services employees [6]. Other studies have shown that managers and professionals report more occupational sitting time than technicians and trade workers, but that leisure sitting time is similar in these employee groups [7,8]. Hours of paid work may also be influential, as one study has shown that women who work part-time sit less during the whole day than those who work full-time [9]. There is also some evidence that more time spent sitting at work is associated with more time sitting at home, as shown by a study that found female University employees who sat most at work, also sat most for television viewing, on work and non-workdays [10]. As most studies to date have involved small samples in selected workplaces, larger studies are required to clarify the relationships between work hours and sitting time in different occupational groups.

A recent review has examined associations between occupation and physical activity (PA). It found strong associations of leisure time physical activity with occupation type, with higher leisure-time MVPA in professional than in blue collar occupations in most studies, although findings were mixed. In the same review there was a negative association between hours worked and leisure time MVPA [11]. The authors stressed that confounding factors such as hours of work, work demands, and work-related physical activity were often not accounted for [11]. The issue of whether leisure-time PA is influenced or compensated by occupational activity is unclear. In a study of office, call centre and customer service employees in Australia, all employees engaged in more sedentary behaviour and less light intensity activity on work days than non-work days [6] but MVPA was higher on work days than non-work days [6]. Conversely, another study showed that MVPA in office workers was very similar on work and non-work days [12]. The amount of leisure-time MVPA may also be influenced by the physical demands of work; however, findings have been equivocal, with high workplace PA associated with low [7] or high levels of non-occupational PA [11], while other studies showed no difference [13]. These mixed results suggest that the relationship between occupation and non-work activity is currently unclear and should be investigated. 

There is also evidence to suggest that occupational factors may impact sleep quality and duration. Data from the 2009 U.S. Behavioural Risk Factor Surveillance System showed that adults who worked more than 40 h per week were 65% more likely to report insufficient sleep (<7 h per day) than those who worked less than 40 h per week [14] and that those working in the manufacturing sector were more likely than people in the general working population to sleep less than 6 h per night [14]. Working night shifts has also been associated with short sleep duration, as one study found that 44% of night shift workers report sleeping less than 6 h per day compared with 30% of day shift workers [14]. Thus, sleep duration may, not surprisingly, differ according to level and type of employment.

While it is apparent that patterns of sitting, sleeping and being active vary markedly across groups in different work settings, these patterns may be even more complex in women. This is because women are more likely to juggle paid and unpaid work, Australian Bureau of Statistics data show more women work part-time than men, and that women spend more time in household duties than men of the same work status (part-time or full-time) [15]. There is also some evidence that women’s health may be particularly at risk from sedentary behaviour, as shown by associations of television viewing with metabolic syndrome [16] and all-cause mortality [17] in women, but not in men. Finally, the occurrence and nature of sleep disorders appears to be different in women and men [18,19] and gender differences in achieving 150 min/week of MVPA have also been noted [11,20]. 

Time spent sleeping, active and sedentary are mutually exclusive but as time in a day is finite these are likely to compete with or influence each other, particularly on work days where work tasks involve either active or sedentary behaviours. To date, no research has assessed whether time spent sitting, sleeping, and being physical active, differs among women in different occupational groups. Given that there are now more women in the paid workforce [21], it is important to understand patterns of sitting, sleep and PA in women, so that appropriate workplace health interventions can be developed. Data from the Australian Longitudinal Study on Women’s Health provide a unique opportunity to examine how occupation and work hours are associated with time spent in these three key health behaviours in a large population cohort of young and mid-aged working women. It is hypothesised that women in different occupations and with varied paid work hours may have different patterns of sleep, PA and sitting on work days, but that on non-work days their patterns will be similar.

## 2. Methods

### 2.1. Participants

The Australian Longitudinal Study on Women’s Health (ALSWH) is a prospective cohort study of women’s physical and mental health, psychosocial aspects of health (such as socio-demographic and lifestyle factors) and use of health services [22]. The overall aim of the study is to examine the relationships between these factors and to inform governments on implications for health policy and practice. In 1996, three cohorts of women, young (born 1973–1978, aged 18–23 years; *n* = 14,247), mid-aged (born 1946–1951, aged 45–50 years; *n* = 13,715) and older (born 1921–1926, aged 70–75 years; *n* = 12,432), were recruited. The sample was randomly drawn from the Australian national Medicare health insurance database which includes all Australian citizens and permanent residents [23]. The women completed a mailed survey every three years. The study has ethical approval from the Universities of Queensland and Newcastle Ethics Committees, and informed consent was received from all respondents.

Data (cross-sectional) for this paper were taken from the 2009 survey of the young cohort and the 2010 survey of the mid-aged cohort, as these surveys included questions on domain specific sitting time and sleep duration. Participants were included if they reported being employed and answered the questions on domain specific sitting time, sleeping time, PA engagement and demographic variables included in the analyses (see the Supplemental Figure S1 for a flow chart of participant numbers). As the focus is on working women, data from the older cohort were not included.

### 2.2. Measures

#### 2.2.1. Outcome Measures

Participants were asked to report the time (hours and minutes) spent sitting on their most recent work and non-work day, separately for work, transport, television viewing, leisure-time computer use and all other purposes. Times spent in all domains were summed separately for work days and non-work days to give overall sitting time per work day and non-work day. These questions were based on the validated questionnaire developed by Marshall et al. [24]. Participants with data for total sitting time of greater than 24 h/day was not included in the analyses. Participants were also asked to report their sleep duration (hours and minutes) for the most recent work and non-work day. Data for participants who reported sleeping for greater than 24 h/day was not included in the analyses. Sleep duration was reported as a continuous variable (h/day) and categorised as shorter (≤6 h/day compared with >6 h/day) or longer sleep (≥8 h/day compared with <8 h per day) duration on both work days and non-work days. Sleep cut points were based on literature suggesting harmful effects of sleeping for 6 h or less and for 8 h or more [1,2,25]. PA was measured using the Active Australia Questionnaire [26], which asks participants to report the frequency and duration (hours) of brisk walking and moderate and vigorous intensity activity for transport or leisure in the last week in bouts of 10 min or more. PA was categorized as meeting guidelines or not, using a PA score of MET (metabolic equivalent) minutes/week, calculated as the sum of the products of total weekly minutes in each of the three categories and their generic MET values (walking minutes × 3.0 METs) + (moderate-intensity PA minutes × 4.0 METs) + (vigorous intensity PA minutes × 7.5 METs). A score of ≥600 was used to denote meeting current PA guidelines (≥150 min of moderate-intensity activity per week) [26].

#### 2.2.2. Predictors

Participants reported their main occupation in the following categories: (a) manager or administrator; (b) professional; (c) associate professional; (d) tradesperson or related worker; (e) advanced clerical or service worker; (f) intermediate clerical, sales or service worker; (g) intermediate production or transport worker; (h) elementary clerical, sales or service worker; or (i) laborer or related worker. These categories were collapsed into manager or professional (a, b and c); clerical or sales (e, f or h) and trades, transport or laborer (d, g or i). Participants reported the average number of hours worked per day; responses were categorized as part-time (<35 h/week) or full-time (≥35 h/week) under the heading of ‘work hours’. They also reported if their work was shift, night, casual, from home, self-employment or in more than one job (not mutually exclusive). ‘Work pattern’ was categorized as “shift or night” if participants reported they worked either shifts or nights; and all others were categorized as “not shift or night”.

#### 2.2.3. Covariates

Area of residence, highest education level, marital status, number of children ≤16 years, smoking, alcohol intake, self-rated health, height and weight were self-reported. Body mass index (BMI; kg/m^2^) was calculated using self-reported weight and height and classified using the World Health Organization categories [27].

### 2.3. Statistical Analysis

Analyses were conducted in 2015 using SPSS version 22.0 (IBM Corporation, Armonk, NY, USA) with statistical significance set at *p* < 0.01 (two-tailed). Mean work hours, domain specific sitting time and overall sitting, and sleep variables were calculated separately for occupation (manager/professional, clerical/sales, trades/production/labourer), work hours (full or part-time) and work pattern (night/shift or regular hours) categories. Differences in mean values of sitting and sleep among groups of occupation, work hours and work pattern were tested using analysis of variance (ANOVA), adjusting for variables that were significantly associated with work variables and sitting or sleep in univariate analyses. Differences between time spent in workday and non-workday sitting and sleeping were assessed using ANOVA, with adjustment for demographic variables associated with workday and non-workday sitting or sleep (BMI, children (young cohort)), marital status, area of residence, education, smoking, alcohol intake and other work categories). Two-way ANOVAs were conducted to examine the interaction effect of working eight hours a day or more with occupation, work hours and work pattern on sitting time and sleeping on workdays. Time spent sitting in specific domains was reported as median (25th and 75th percentile). Physical activity hours per week were reported as medians (interquartile range) for each category of occupation, work hours and work pattern, with differences between categories tested using Kruskal-Wallis tests. The odds of reporting: (1) short and long sleep duration (≤6 h/day and ≥8 h/day) and (2) meeting physical activity guidelines, in categories of occupational, work hours and work pattern regularity variables, were examined using logistic regression models, both unadjusted and with adjustment for variables that were significantly associated with work variables, sitting time, sleep and PA (i.e., BMI, number of children (young cohort only), marital status, area of residence, education, smoking, alcohol intake, self-rated health and other work categories). Only findings significant at the 0.01 level in adjusted analyses were discussed in text. Data were included for participants who were working, answered sitting, sleeping and physical activity questions with realistic values and had demographic data of interest in the 2009 young cohort survey and 2010 mid aged cohort survey. A flow chart for inclusion is included in Supplementary Data Figure S1.

## 3. Results

The sociodemographic characteristics of the young and mid-aged women, separated into categories for occupation, work hours and work pattern, are presented in Table 1. Women in the three occupational groups were significantly different according to all measured characteristics in the young cohort, and all except BMI and alcohol intake in the mid-aged cohort. Similarly, women who worked part-time were different from those who worked full-time, on all measured characteristics in the young cohort and all except smoking, children under 16 years living at home and alcohol intake in the mid-aged cohort. Women who worked regular hours were more likely to be married and had a lower BMI than shift or night workers in both the young and mid-aged cohorts, and were less likely to have a post-high school qualification and be a current smoker in the mid-aged cohort only.

Findings are presented for adjusted models except where unadjusted models showed disparate findings.

### 3.1. Work Hours

Self-reported hours worked on work days, sleep (work and non-work days), sitting (work and non-work days) and MVPA are presented in Table 2. Hours spent at work on work days differed among all occupational, work hours and work pattern categories in both the young and mid-age cohorts; manager/professionals worked longer hours than clerical/sales and trades/production/ laborer (not in young cohort), full-time longer than part-time and shift/night longer than those who worked regular hours.

### 3.2. Sitting Time

There were differences in sitting time on work days and non-work days in occupational categories, with sitting being greater on work days than non-work days for manager/professionals, clerical/sales, full-time workers, and those working regular hours, and less for trades/production/laborers and shift or night workers (compared with alternate categories, Table 2). This pattern was consistent for both the young and mid-aged cohort. Part-time workers in the mid-aged cohort reported less sitting on work days than non-work days but there was no difference between these days in the young cohort. Differences between sitting time in various groups were mainly seen on work days, with trades/production/laborers sitting less on work days than clerical/sales and manager/professionals (differences around 3–4 h); shift/night workers sitting less than those working regular hours, and full-time workers sitting more than part-time, in both cohorts. There was an interaction between working longer hours and full-time/part-time status and with regular/shift night for the young cohort but not for the mid-aged cohort. In the young cohort those who work full time and those who work regular hours and work longer than 8 h/day reported longer sitting times. There was no interaction between working eight hours or more and occupation, with sitting longer for either cohort.

**Figure 1:**
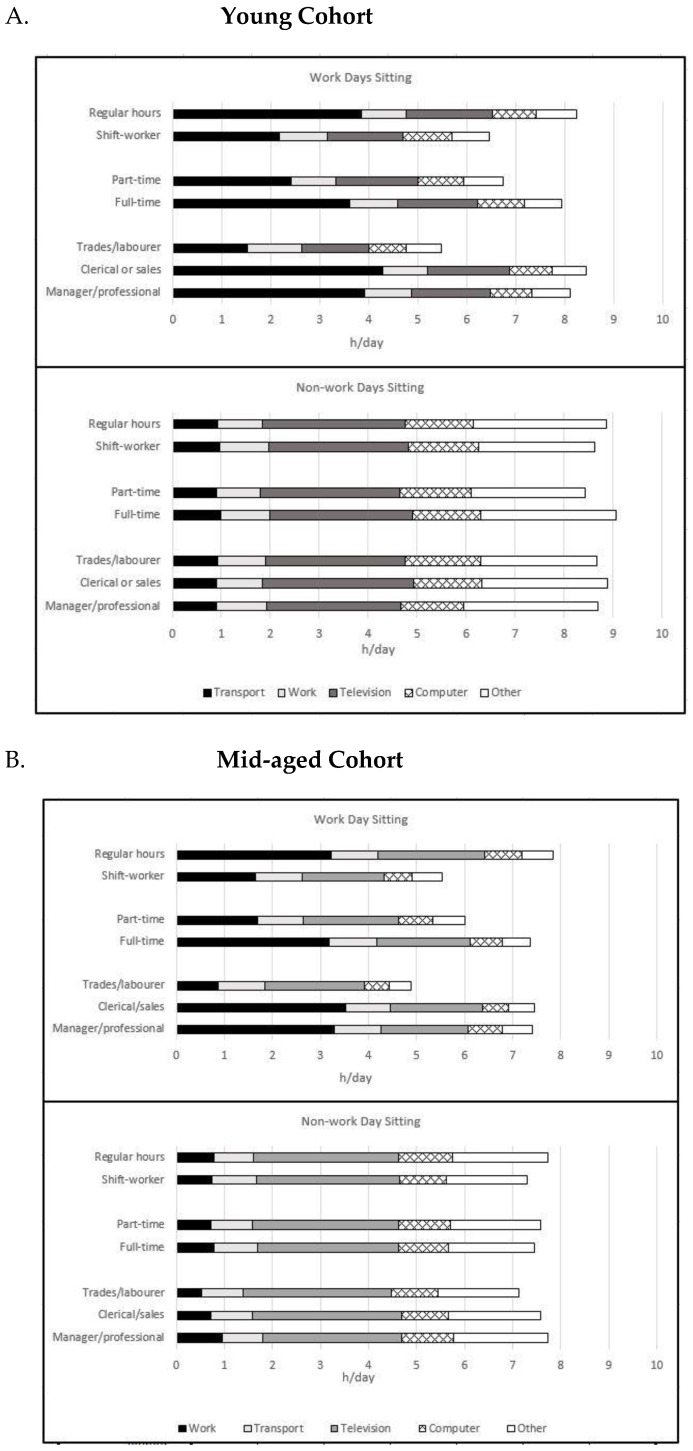
Time (h/day) reported for the individual sitting time questions on work days and non-work days by (**A**) young and (**B**) mid-aged women. Adjusted for marital status, living with children (young cohort only), education, work hours, body mass index, drinking, smoking, area of residence and other work factors. Young cohort: in 2009 aged 31–36 years (*n* = 4650); Mid-aged cohort: in 2010 aged 59–64 years (*n* = 3185).

Figure 1 shows the time spent sitting in specific domains. The main differences between work categories were in sitting for work on work days. Unsurprisingly, in both cohorts those who worked regular hours (compared with night/shift workers), full-time (compared with part-time) and were managers/professionals or sales clerical workers (compared with trades/production/laborers) sat more for work on work days. This difference was between two and six hours per day. Times reported for individual domain specific items are included in Supplementary Table S1.

### 3.3. Sleep

For all groups, sleep duration was about half an hour longer on non-work days than work days. On work days, shift/night workers slept less than regular workers in both cohorts; in the young cohort, trades/production/laborers slept less than other occupational categories and in the mid-aged cohort full-time workers slept less than part-time. On non-work days, full-time workers slept more than part-time workers in both cohorts and in the young cohort trades/production/laborers slept less than manager/professionals. Interactions between working eight hours a day or more and work characteristics, with sleeping were not significant. In the young cohort, the prevalence of shorter and longer sleep was 6.2% and 12.4% respectively on workdays and 2.8% and 35.9% respectively on non-work days. Similarly, in the mid-age cohort, the prevalence of shorter and longer sleep was 10.1% and 9.3% respectively on work days and 5.8% and 22.4% respectively on non-work days in the mid-aged cohort. The odds of reporting shorter and longer sleep are presented in Table 3. The odds of reporting shorter sleep duration on workdays were higher in shift/night workers than regular hours workers, for both the young and mid-aged cohorts.

### 3.4. Physical Activity

Time spent in MVPA also differed among various work characteristic groups and in the young and mid-aged cohorts. In the young cohort, clerical/sales workers reported less MVPA than manager/professionals and trades/production/laborers; and full-time workers reported more MVPA than part-time workers. In the mid-aged cohort, trades/production/laborers reported more MVPA than clerical/sales and manager/professionals; and full-time workers reported less MVPA than part-time workers. The odds of achieving PA guidelines are presented in Table 4. In adjusted analyses, the odds of achieving guidelines were lower for clerical/sales workers (compared to manager/professionals) in the young cohort and for full-time workers (compared to part-time) in the mid-aged cohort. There was a difference between the adjusted and unadjusted findings for the odds of achieving guidelines in the young cohort, when examining by work status. In adjusted models, there was no longer a significant difference between those who work part-time or full-time; the main modifier was having children or not.

## 4. Discussion

The workplace is an opportune setting for health promotion programs, which aim to improve employee health status and productivity [28]. Given that many women are now in the paid workforce [29], and are juggling their work with unpaid family and caring responsibilities [15], it is likely that time pressure might impact on the time available for sleep and physical activity. As many women are also in occupations such as office work, which require them to sit for long hours [6,30], they may be exposed to a trifecta of behavioural risk factors, too much sitting, too little sleep and insufficient physical activity. This study examined how these behaviours vary in women with different occupations and work patterns, as improved understanding of the patterns of these behaviours in working women could help to identify ‘at risk’ groups for health promotion intervention. Differences in self-reported sitting time, sleep and PA were found between groups of women with different work characteristics in both the young and mid-aged cohort. As hypothesised, these differences occurred on work days, particularly. Notably on work days, full-time workers were most likely to report high sitting time, as were those in the managerial/professional or clerical/sales occupations. Work days were, however, different from non-work days in many ways. Not surprisingly, sleep durations were lower on work days than non-work days, most notably amongst those who worked shifts or nights, but also in full-time workers across occupational groups. Physical activity was not reported separately for work and non-work days, but over the week. Nonetheless, work related differences were still noted, with mid-age full-time workers and younger clerical/sales workers least likely to meet the PA guidelines. 

### 4.1. Sitting Time

Average sitting times were comparable with other Australian data [31] yet higher than those reported internationally [32]. The higher levels of sitting reported in the current study are likely to reflect the multiple domain measure, which provides estimates that are about 2 h higher (per day) than when a single item sitting estimate, such as the International Physical Activity Questionnaire is used [33]. On workdays, differences in sitting time followed expected patterns in both the young and mid aged cohorts; managers/professionals and clerical/sales workers, full time workers and regular pattern workers reported higher durations of sitting time, as has been previously shown [31,34,35] and this appears to be driven by high work sitting on work days (Figure 1). The amount of sitting time reported by younger women who work full-time with regular hours appeared to be particularly influenced by working longer hours, which often equate with more work-based sitting [36]. Non-work day sitting did not differ among any of the work-related groups, even for those who sat less on work days (trades/production/labourers). Differences in non-work day sitting have not been consistently observed in previous studies [31,34]. It appears that differences in sitting time in employed women are largely due to sitting for work, although this should be confirmed by studies using objective measures of sitting time.

### 4.2. Sleep

In both cohorts, average sleep duration was consistent with previous Australian reports [37,38], which have shown that shift-work is typically associated with shorter sleep durations and poorer sleep quality [39]. This was borne out by our findings that shift and night workers in both cohorts were mostly likely to report shorter sleep on work days. In both cohorts, average sleep times were longer on non-work days than workdays, which may be a strategy for trying to catch up on sleep.

### 4.3. Physical Activity

Previous reviews have identified that blue collar workers report higher levels of occupational PA and lower levels of leisure time PA than white collar and professional workers [11,40]. However, in the current study, after adjusting for covariates, it was not the ‘blue collar’ (used to describe the trades/production/labourer category in this study) workers, but the clerical/sales workers, who were significantly less likely to be physically active, when compared with managers/professionals in the young cohort. This finding is consistent with a previous report which found that managers/professionals were more likely to be active than other occupational groups, in women of similar age [41]. In the mid-aged cohort, there was no association between occupational category and physical activity, although full-time workers were less likely (than part-time) to achieve physical activity recommendations. This might be expected as there is potentially more leisure-time available with part-time work. As the nature of the association between occupational category and physical activity is inconsistently observed in other studies [7,11,13], it may be that life-stage is important. Even the mid-age women who worked full time were surprisingly active; this may reflect their lesser involvement in child care and household work than the younger women. Others have reported that MVPA declines with age [20], but previous ALSWH reports have shown that women tend to increase their activity levels when children leave home [42]. The presence of young children may be a factor in the differences noted in achieving physical activity guidelines between the young and mid-aged cohorts. In contrast to the mid aged cohort, full-time workers in the young cohort were more likely to achieve guidelines in unadjusted analyses, but this finding was diminished after adjustment for demographic variables. This may have been due to the fact that part-time workers in the young cohort were more likely to have children in the household (85% compared with 30% for full-time workers; Table 1). Previous research has shown that having young children decreases participation in physical activity [43,44].

### 4.4. Similarities and Differences between Cohorts

In terms of which work groups reported more sitting, short or long sleep, and not meeting physical activity guidelines, the two cohorts were remarkably similar. For both cohorts, manager/professionals and clerical/sales workers reported more sitting than trades/labourers, full-time more than part-time and those who worked regular hours more than shift/night workers. For sleep, those working shifts or at night were more likely to report short sleep on work days in both cohorts. Clerical/sales workers in the young cohort and the full-time workers in the mid-aged cohort were less likely to achieve physical activity guidelines. Interestingly, both these groups also reported higher sitting times, so it is possible that these groups are at even greater health risk as high sitting time and low physical activity have detrimental effects on health and mortality [45]. Patterns of sleeping and sitting across work and non-work days were also similar in the two cohorts. For most groups, participants reported sitting less and sleeping longer on non-work days than work days. It is possible that away from the restrictions imposed by work conditions and weekday schedules that women engage in less sitting time and attempt to increase sleep duration on non-work days. However, attempts to catch up sleep on non-work days by sleeping for longer may also present other challenges to maintaining a healthy sleep schedule and wellbeing [46]. Twenty-four-hour data collection would clarify the very variable movement and sleep patterns on non-work and work days.

Several limitations should be acknowledged when interpreting the findings of this study. As with all cohort studies, there has been attrition over time, so that the included women were less representative of the general population than the starting cohort in 1996 and particularly in the young cohort, there is a bias towards more educated women [47]. However, the very large sample size and inclusion of women from across all occupational categories and work patterns is a strength. Another limitation is that all the data were self-reported, though the measures of PA and sitting have acceptable psychometric properties. In relation to sleep, we did not include measures of sleep quality, timing, chronotype or presence of sleep disorders. As the study only examines the associations cross-sectionally we cannot infer causality.

The categories used for occupation type were broad and this may lead to some dilution or masking of effects. For example, clerical, sales and retail were collapsed into one category; however, we know that office workers (likely to include clerical workers) sit for longer at work than retail workers [6].

While most of the findings reported here appear to reflect ‘conventional thinking’, they do provide insight for targeting public health campaigns. Occupational characteristics were associated with higher sitting, shorter sleep and less activity, suggesting an important role for workplaces in promoting healthier behaviours. Differences in sitting time are largely driven by sitting for work and those groups that experience high sitting time at work (manager/professionals, clerical/sales and full-time workers) should be targeted. Workplace modifications including sit-stand desks have been shown to be effective at reducing workplace sitting [48]. Those at health risk from short sleep are the shift and night workers, who may benefit from cognitive behavioural sleep programs [49,50]. The groups at risk from low physical activity varied in the two cohorts. The combination of low physical activity and high sitting time in young clerical sales workers and mid-aged full-time workers was of particular concern. These workers could benefit from a two-pronged approach to increase physical activity and decrease sitting time, perhaps replacing one behaviour with the other, which has been shown to have a beneficial effect in studies that used 24 h monitor data [51].

## 5. Conclusions

These results indicate that the patterns, amounts and types of women’s paid work impact on three key health behaviours, which are known to have long term effects on the development of a range of chronic diseases. If women are to remain sufficiently healthy to stay in the workforce as they age, it will be important to identify groups who are most ‘at risk’ of poor health behaviours and develop tailored approaches for prevention. Those most at risk of either too much sitting, too little sleep, too little physical activity or a combination of these appear to be women who work shifts and nights, full-time hours, and those in clerical/sales and managerial roles. These results suggest multiple behaviour interventions tailored to work patterns and occupational category may be useful to improve sleep, sitting and activity behaviours in working women.

## Figures and Tables

**Table 1:** Demographic characteristics of the young (*n* = 4650) and mid-aged (*n* = 3185) women by occupational category, work hours and work patterns; Australian Longitudinal study on Women’s Health (ALSWH).

	Occupational Category				Work Hours			Work Pattern		
	Manager/Professional	Clerical/Sales	Trades/Production/Labourer	*p*	Full-Time	Part-Time	*p*	Regular Hours	Shift or Night Worker	*p*
Young cohort										
*N*	2986	1370	294		2688	1962		3889	761	
Age in years, mean (SD)	33.6 (1.5)	33.8 (1.5)	33.9 (1.5)	<0.001	33.6 (1.5)	33.8 (1.4)	<0.001	33.7 (1.5)	33.6 (1.5)	0.51
Area of residence urban (%)	70	56	41	<0.001	68	58	<0.001	64	62	0.14
Post High school qualification (%)	95	64	57	<0.001	86	79	<0.001	83	83	0.99
Married or in de-facto relationship (%)	73	75	74	0.47	64	88	<0.001	74	71	0.07
One or more children under 16 years in house (%)	47	62	67	<0.001	30	85	<0.001	53	53	0.67
Current smoker (%)	12	16	26	<0.001	16	11	<0.001	13	15	0.17
Risky drinker ^a^ (%)	5	6	9	<0.01	6	4	<0.01	5	5	0.53
Body Mass Index kg/m^2^ (mean, SD)	25.2 (5.4)	26.7 (6.4)	26.6 (5.8)	<0.001	25.9 (6.0)	25.5 (5.5)	<0.001	25.6 (5.8)	26.7 (6.4)	<0.001
Self-rated health good to excellent (%)	93	90	87	<0.001	92	92	0.63	92	91	0.31
Work characteristics										
Full-time workers (%)	65	47	39	<0.001				58	56	0.15
Work shift of night (%)	16	14	26	<0.001	16	17	0.15			
Manager or professional (%)					72	54	<0.001	64	64	<0.001
Clerical or sales (%)					24	37		30	26	
Trade, production or labourer (%)					4	9		6	10	
Mid-aged cohort										
*N*	1724	1121	340		1443	1742		2723	462	
Age in years, mean (SD)	61.2 (1.4)	61.2 (1.4)	61.4 (1.4)	0.04	61.1 (1.4)	61.3 (1.4)	0.24	61.2 (1.4)	61.2 (1.3)	0.03
Area of residence urban (%)	44	43	30	<0.001	45	40	<0.01	43	43	0.95
Post High school qualification (%)	77	33	25	<0.001	59	54	0.02	55	63	<0.01
Married or in de-facto relationship (%)	75	75	78	0.55	70	80	<0.001	77	68	<0.001
One or more children under 16 years in house (%)	1	3	2	0.01	2	2	0.82	2	3	0.12
Current smoker (%)	7	7	12	0.01	8	7	0.55	7	12	<0.001
Risky drinker ^a^ (%)	7	6	8	0.62	8	6	0.11	7	5	0.19
Body Mass Index kg/m^2^ (mean, SD)	26.9 (5.4)	27.1 (5.2)	26.9 (5.4)	0.61	27.2 (5.5)	26.8 (5.1)	0.03	26.8 (5.2)	27.6 (5.5)	0.15
Self-rated health good to excellent (%)	93	93	89	0.03	93	94	0.73	93	91	0.10
Work characteristics										
Full-time workers (%)	54	36	32	<0.001				45	46	0.71
Work shift of night (%)	16	12	17	<0.01	15	14	0.72			
Manager or professional (%)					45	65	<0.001	53	58	<0.01
Clerical or sales (%)					41	28		36	30	
Trade, production or labourer (%)					13	8		10	13	

Young cohort: in 2009 aged 31–36 years; Mid-aged cohort: in 2010 aged 59–64 years; ^a^: three or more standard drinks per day; *N*: number; SD: standard deviation; *p*: level of significance.

**Table 2:** Total sleep, sitting and physical activity duration on work and non-work days reported by young (*n* = 4650) and mid-aged (*n* = 3185) women in the ALSWH.

Work Characteristic	Work Hours Per/Workday	Sleep			Sitting Time			Moderate to Vigorous Physical Activity (h/week) Median (25%, 75%)
		Work Day (h/day)	Non-Work Day (h/day)	*p* Work/Non-Workday	Work Day (h/day)	Non-Work Day (h/day)	*p* Work/Non-Workday	
Young Cohort								
Total Sample	8.25 (2.43)	7.31 (1.14)	8.04 (1.27)	<0.001	8.68 (4.18)	8.18 (3.85)	<0.001	2.5 (1, 4.50)
Occupational category								
Manager/professional	8.53 (2.25)	7.32 (1.10)	8.07 (1.22)	<0.001	8.99 (4.08)	8.20 (3.82)	<0.001	2.67 (1, 5)
Clerical/sales	7.75 (2.48) ^a^	7.34 (1.22)	8.00 (1.33)	<0.001	8.76 (4.09)	8.21 (3.89)	<0.001	2.00 (0.75, 4) ^a^
Trades/production/labourer	7.76 (3.41)	7.16 (1.31) ^b^	7.87 ^a^ (1.44)	<0.001	5.06 (3.93) ^a,b^	7.93 (3.98)	<0.001	2.50 (0.5, 5) ^b^
Work hours								
Part-time	7.49 (3.08)	7.35 (1.20)	7.78 (1.25)	<0.001	7.28 (3.97)	7.31 (3.59)	0.47	2.00 (1, 4)
Full-time	8.82 (1.59) ^a^	7.29 (1.11)	8.22 (1.25) ^a^	<0.001	9.69 (4.05) ^a^	8.82 (3.91) ^a^	<0.001	2.75 (1, 5) ^a^
Work pattern regularity								
Not shift or night worker	8.17 (2.39)	7.37 (1.11)	8.05 (1.26)	<0.001	8.98 (4.06)	8.20 (3.84)	<0.001	2.5 (1, 4.5)
Shift or night worker	8.65 (2.60) ^a^	7.02 (1.30) ^a^	7.97 (1.32)	<0.001	7.11 (4.43) ^a^	8.12 (3.94)	<0.001	2.5 (1, 5)
Mid-aged Cohort (aged 59–64 years)								
Total Sample	7.48 (2.55)	7.06 (1.22)	7.57 (1.29)	<0.001	7.59 (4.11)	7.57 (3.74)	0.20	3.50 (1.50, 7)
Occupational category								
Manager/professional	7.87 (2.41)	7.05 (1.23)	7.61 (1.28)	<0.001	7.99 (4.05)	7.74 (3.79)	0.05	3.50 (1.50, 6.54)
Clerical/sales	7.10 (2.45) ^a^	7.10 (1.17)	7.54 (1.27)	<0.001	7.81 (4.06)	7.50 (3.68)	0.02	3.50 (1.17, 6.21)
Trades/production/labourer	6.71 (3.18) ^a,b^	6.95 (1.36)	7.48 (1.44)	<0.001	4.86 (3.53) ^a,b^	6.95 (3.64) ^a,b^	<0.001	4.00 (1, 7) ^a,b^
Work hours								
Part-time	6.74 (2.84)	7.11 (1.31)	7.51 (1.34)	<0.001	6.80 (3.97)	7.54 (3.72)	<0.001	4.00 (1.81, 7)
Full-time	8.42 (1.73) ^a^	7.00 (1.11) ^a^	7.65 (1.20) ^a^	<0.001	8.56 (4.06) ^a^	7.60 (3.76)	<0.001	3.00 (1, 6) ^a^
Work pattern regularity								
Not shift or night worker	7.37 (2.53)	7.11 (1.19)	7.57 (1.28)	<0.001	7.90 (4.06)	7.60 (3.71)	<0.001	3.50 (1.50, 7)
Shift or night worker	8.18 (2.55) ^a^	6.75 (1.37) ^a^	7.55 (1.33)	<0.001	5.78 (3.95) ^a^	7.43 (3.89)	<0.001	3.00 (1, 6.5)

Data are mean (standard deviation) except where indicated. Testing for differences in mean values are adjusted for body mass index (BMI), number of children, marital status, area of residence, education, smoking, alcohol intake, self-rated health, work hours per day and other work categories ^a^: different to top category; ^b^: different to second category.

**Table 3:** Odds ratios (and 95% CIs) for reporting short and long sleep duration young (*n* = 4650) and mid-aged (*n* = 3185) women in the ALSWH.

	Young Cohort				Mid-Aged Cohort			
	Work Day		Non-Work Day		Work Day		Non-Work Day	
	Shorter Sleep	Longer Sleep	Shorter Sleep	Longer Sleep	Shorter Sleep	Longer Sleep	Shorter Sleep	Longer Sleep
	≤6 h/Night (*n* = 289)	≥8 h/Night (*n* = 578)	≤6 h/Night (*n* = 128)	≥8 h/Night (*n* = 1671)	≤6h/Night (*n* = 322)	≥8 h/Night (*n* = 297)	≤6 h/Night (*n* = 184)	≥8 h/Night (*n* = 715)
Occupational category								
Manager/professional	Ref	Ref	Ref	Ref	Ref	Ref	Ref	Ref
Clerical/sales	0.98 (0.71, 1.34)	1.32 (1.06, 1.64)	1.11 (0.71, 1.76)	1.22 (1.04, 1.43)	1.03 (0.77, 1.39)	0.71 (0.52, 0.98)	1.18 (0.81, 1.73)	0.97 (0.79, 1.20)
Trades/production/labourer	1.38 (0.86, 2.22)	1.12 (0.75, 1.67)	1.31 (0.63, 2.72)	1.20 (0.90, 1.60)	1.04 (0.68, 1.59)	0.76 (0.48, 1.20)	1.07 (0.62, 1.85)	0.99(0.72, 1.36)
Work hours								
Part-time	Ref	Ref	Ref	Ref	Ref	Ref	Ref	Ref
Full-time	1.01 (0.74, 1.38)	0.76 (0.60, 0.95)	1.21 (0.77, 1.89)	1.21 (1.02, 1.43)	1.10 (0.86, 1.41)	0.63 (0.48, 0.82)	0.81 (0.58, 1.12)	1.21 (1.01, 1.44)
Work pattern regularity								
Regular hours	Ref	Ref	Ref	Ref	Ref	Ref	Ref	Ref
Shift or night worker	1.94 (1.46, 2.58) **	0.77 (0.59, 1.01)	1.01 (0.61, 1.68)	0.88 (0.74, 1.05)	2.03 (1.51, 2.71) *	0.60 (0.39, 0.93)	0.88 (0.55, 1.41)	0.88 (0.68, 1.13)

Analyses adjusted for BMI, children (young cohort), marital status, area of residence, education, smoking, alcohol intake, self-rated health and other work categories. Shorter sleep compared to >6 h/night, longer sleep compared to <8 h/night. *: *p* < 0.01; **: *p* < 0.001; CI: confidence interval; Ref: reference category.

**Table 4:** Odds of young (*n* = 4650) and mid-aged (*n* = 3185) women reporting meeting physical activity guidelines in the ALSWH.

	Young		Mid-Aged	
	Unadjusted	Adjusted	Unadjusted	Adjusted
Occupational category				
Manager/professional	Ref	Ref	Ref	Ref
Clerical/sales	0.67 (0.60, 0.76) **	0.76 (0.66, 0.88) **	1.09 (0.95, 1.26)	0.93 (0.78, 1.11)
Trades/production/labourer	0.80 (0.64, 1.01)	1.01 (0.77, 1.31)	1.01 (0.81, 1.26)	1.08 (0.83, 1.40)
Work hours				
Part-time	Ref	Ref	Ref	Ref
Full-time	1.46 (1.31, 1.63) **	0.96 (0.83, 1.12)	0.76 (0.67, 0.86) **	0.78 (0.67, 0.91) *
Work pattern regularity				
Regular hours	Ref	Ref	Ref	Ref
Shift or night worker	0.94 (0.81, 1.09)	0.92 (0.78, 1.09)	0.89 (0.74, 1.07)	0.93 (0.76, 1.15)

Adjusted analyses included BMI, marital status, children <16 years in household (young cohort), area of residence, education, smoking, alcohol intake, self-rated health and other work categories. *: *p* < 0.01; **: *p* < 0.001; Ref: reference category.

## Supplementary Materials

The following are available online at www.mdpi.com/1660-4601/14/3/290/s1, Figure S1: Flow Charts for inclusion of participants’ data young and mid-aged women in the Australian Longitudinal Study on Women’s Health. Table S1: Time reported on the individual sitting time questions on work days and non-work days (hours/day) in young (*n* = 4650) and mid-aged (*n* = 3185) women in the Australian Longitudinal Study on Women’s Health (ALSWH).
