# Supplementary material for: Sitting Time, Physical Activity and Sleep by Work Type and Pattern—The Australian Longitudinal Study on Women’s Health
Source: Int J Environ Res Public Health. 2017 Mar 10;14(3):290. doi: 10.3390/ijerph14030290 (PMC5369126; doi:10.3390/ijerph14030290)
Supplement: Supplementary file 1 [file ijerph-14-00290-s001.pdf]

Supplementary Materials: Sitting Time, Physical Activity and Sleep by Work Type and Pattern—The Australian Longitudinal Study on Women's Health

Bronwyn K. Clark, Tracy L. Kolbe-Alexander, Mitch J. Duncan and Wendy Brown

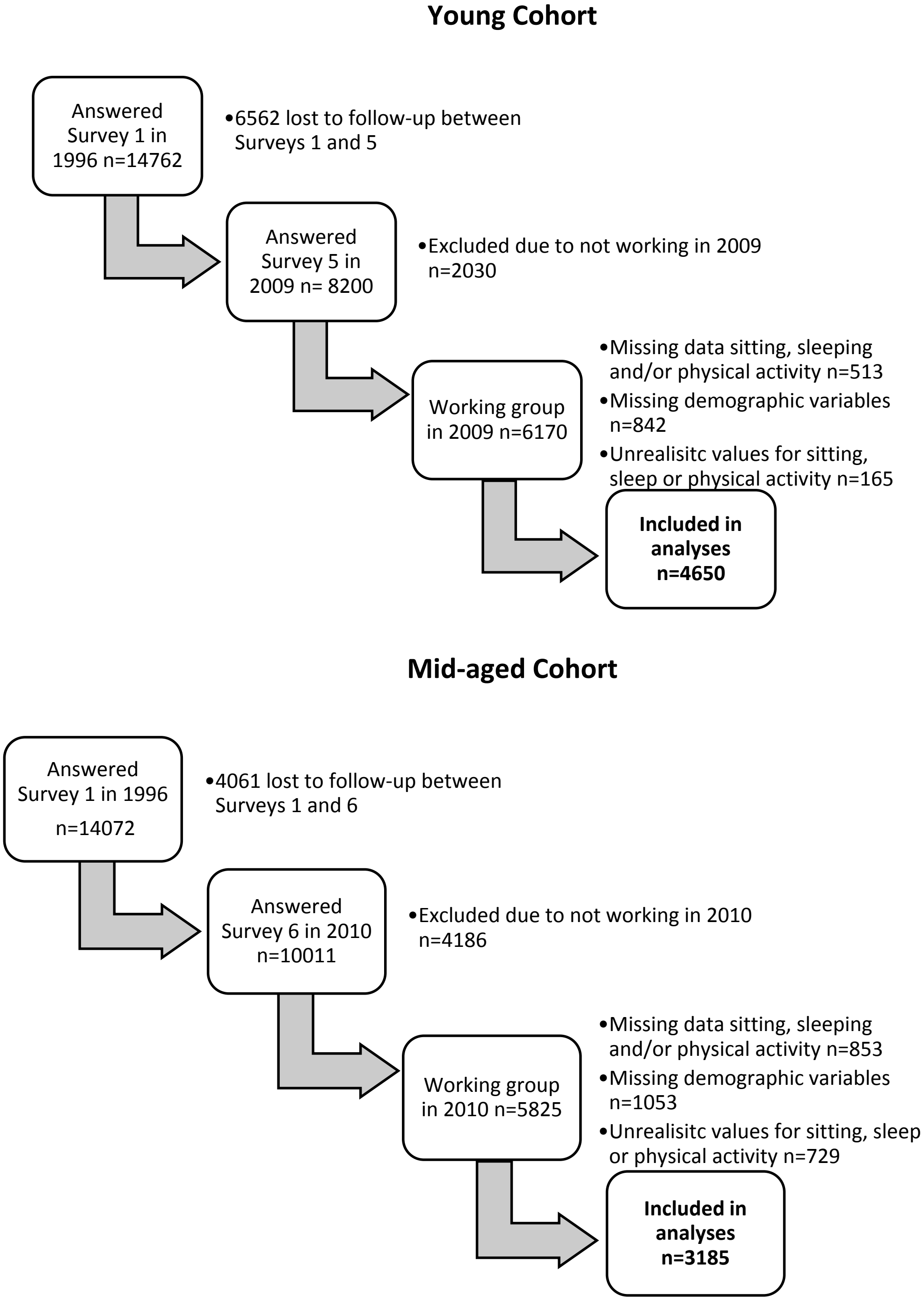

Figure S1. Flow Charts for inclusion of participants' data young and mid-aged women in the Australian Longitudinal Study on Women's Health.

Table S1: Time reported on the individual sitting time questions on work days and non-work days (hours/day) in young (n = 4650) and mid-aged (n = 3185) women in the Australian Longitudinal Study on Women's Health (ALSWH).

	**Work Days**					**Non-Work Days**				
	Work	Transport	TV	Computer	Other sedentary	Work	Transport	TV	Computer	Other sedentary
Young cohort										
Total Sample	5 (1, 7)	0.75 (0.33, 1.33)	2 (0.75, 2)	0.50 (0, 1)	0 (0, 1)	0 (0, 1)	0.50 (0.33, 1)	2 (2, 4)	1 (0.50, 2)	2 (1, 4)
Occupational category										
Manager/professional	5.50 (2, 7.50)	0.83 (0.50, 1.33)	2 (1, 2)	0.50 (0, 1)	0.17 (0, 1)*	0 (0, 1)	0.50 (0.33, 1)	2 (1.5, 3)	1 (0.50, 2)	2 (1, 4)
Clerical/sales	6 (2, 7)*	0.67 (0.33, 1)*	2 (1, 2)	0.50 (0, 1)	0 (0, 1)	0 (0, 1)	0.50 (0.33, 1)	3 (2, 4)*	1 (0.50, 2)	2 (1, 3)*
Trades/production/labourer	0 (0, 2)*	0.67 (0.33, 1.17)*	1 (0, 2)*	0.33 (0, 1)*	0 (0, 1)*	0 (0, 1)*	0.50 (0.25, 1)	2.50 (1.50, 4)*	1 (0.50, 2)	2 (0, 3)*
Work hours										
Part-time	3 (0.50, 6)	0.67 (0.33, 1)	1.50 (0.50, 2)	0 (0, 1)	0 (0, 1)	0 (0, 1)	0.50 (0.33, 1)	2 (1.5, 3)	1 (0.50, 2)	2 (0.50, 3)
Full-time	6 (3, 8)	0.83 (0.42, 1.50)*	2 (1, 2)	0.50 (0, 1)	0.25 (0, 1)*	0 (0, 1)*	0.67 (0.33, 1)*	2.50 (2, 4)*	1 (0.50, 2)*	2 (1, 4)*
Work pattern regularity										
Regular hours	5.50 (2, 7.5)	0.75 (0.33, 1.33)	2 (1, 2)	0.50 (0, 1)	0 (0, 1)	0 (0, 1)	0.50 (0.33, 1)	2 (2, 4)	1 (0.50, 2)	2 (1, 4)
Shift or night worker	2 (0.50, 6)*	0.67 (0.33, 1.29)	1 (0, 2)	0.50 (0, 1)	0 (0, 1)	0 (0, 1)	0.50 (0.33, 1)	2 (2, 4)	1 (0.50, 2)	2 (0.71, 3)*
Mid-aged Cohort										
Total Sample	2.5 (0.33, 6)	0.5 (0.25, 1)	2 (1, 3)	0.25 (0, 1)	0 (0, 1)	0 (0, 1)	0.5 (0, 1)	3 (2, 4)	0.67 (0, 1.5)	2 (0, 3)
Occupational category										
Manager/professional	3.50 (1, 6)	0.67 (0.33, 1.21)	2 (1, 3)	0.50 (0, 1)	0 (0, 1)	0.25 (0, 1.5)	0.50 (0.17, 1)	3 (2, 4)	1 (0.01, 2)	2 (0.50, 3)
Clerical/sales	4 (0.33, 6.50)*	0.5 (0.08, 1)	2 (1, 3)	0 (0, 0.50)*	0 (0, 1)	0 (0, 1)*	0.50 (0.08, 1)	3 (2, 4)	0.50 (0, 1)*	2 (0, 3)
Trades/production/labourer	0 (0, 0.50)*	0.50 (0.17, 1)*	2 (1, 3)*	0 (0, 0.50)*	0 (0, 0.50)*	0 (0, 0.33)*	0.50 (0, 1)	3 (2, 4)	0.50 (0, 1)*	1 (0, 2.50)*
Work hours										
Part-time	1 (0, 4.50)	0.50 (0.25, 1)	2 (1, 3)	0.33 (0, 1)	0 (0, 1)	0 (0, 1)	0.50 (0, 1)	3 (2, 4)	1 (0, 1.50)	2 (0, 3)
Full-time	5 (1, 7)*	0.67 (0.33, 1.17)*	2 (1, 3)*	0.25 (0, 1)*	0 (0, 1)	0 (0, 1)	0.50 (0.17, 1)	3 (2, 4)	0.50 (0, 1.50)	2 (0, 3)
Work pattern regularity										
Regular hours	3 (0.33, 6)	0.50 (0.25, 1)	2 (1, 3)	0.33 (0, 1)	0 (0, 1)	0.50 (0, 1)	0.50 (0, 1)	3 (2, 4)	0.75 (0, 1.50)	2 (0, 3)
Shift or night worker	1 (0.17, 3)*	0.50 (0.33, 1)	1.50 (0, 2)*	0 (0, 1)*	0 (0, 1)	0 (0, 1)	0.54 (0.17, 1)	3 (2, 4)	0.50 (0, 1.50)*	2 (0, 3)

Data for sedentary times are median (25, 75%); *: median different to top category $p < 0.01$; Young cohort: in 2009 aged 31-36 years; Mid-aged cohort: in 2010 aged 59-64 years.

© 2017 by the authors; licensee MDPI, Basel, Switzerland. This article is an open access article distributed under the terms and conditions of the Creative Commons by Attribution (CC-BY) license (http://creativecommons.org/licenses/by/4.0/).
